# Prostate cancer patients can benefit from 5-alpha-reductase inhibitor treatment: a meta-analysis

**DOI:** 10.7717/peerj.9282

**Published:** 2020-06-01

**Authors:** Tuo Deng, Xueming Lin, Xiaolu Duan, Zihao He, Zhijian Zhao, Guohua Zeng

**Affiliations:** 1 Department of Urology and Guangdong Key Laboratory of Urology, The First Affiliated Hospital of Guangzhou Medical University, Guangzhou, China; 2 Department of Urology, First Hospital of Shanxi Medical University, Taiyuan, China

**Keywords:** Prostate cancer, 5α-Reductase inhibitors, Treatment effect, Meta-analysis

## Abstract

**Background:**

The efficacy and safety of 5α-reductase inhibitors (5ARIs) in treating prostate cancer (PCa) have not been fully determined. We performed a meta-analysis to evaluate the effectiveness and safety of 5ARIs for PCa patients.

**Methods:**

A comprehensive literature search of online databases was conducted to obtain comparative studies exploring the effectiveness and safety of 5ARIs in treating PCa up to October 2019. Summarized odds ratio s (OR s) or hazard ratio s (HR s) were calculated to compare the outcomes between 5ARI and control groups. Our meta-analysis was registered in PROSPERO under number CRD42018109809.

**Results:**

A total of 2,277 patients from 10 studies were included. No significant difference was found in prostate-specific antigen progression between two groups (OR = 0.82, 95% CI [0.52–1.29], *P* = 0.40). However, 5ARI treatment significantly reduced the total progression of PCa (OR = 0.61, 95% CI [0.48–0.77], *P* < 0.0001), especially for patients with local (OR = 0.56, 95% CI [0.44–0.73], *P* < 0.00001) and low-Gleason score (≤7) PCa (OR = 0.63, 95% CI [0.48–0.84], *P* = 0.002). Additionally, 5ARIs also significantly prolonged the progression-free survival time (HR = 0.57, 95% CI [0.34–0.96], *P* = 0.04) for PCa patients. No significant difference was found in the occurrence of PCa recurrence, metastasis, biopsy reclassification, and side-effects between two groups.

**Conclusions:**

Our study suggests that 5ARI treatment can benefit patients with local and low Gleason score (≤7) PCa, especially in delaying the disease progression. More studies with larger sample size and comprehensive study design are still needed to verify our outcomes.

## Introduction

Prostate cancer (PCa) is the second most common cancer and the fifth leading cause of cancer mortality in men all over the world. Approximately 1.3 million new cases and 359,000 associated deaths existed worldwide in 2018 (Bray et al., 2018). In US, the incidence of PCa is highest in cancer and its related death also ranks the second among male patients. Based on the data, 164,690 new cases and 29,430 deaths are estimated to emerge in the following year (Siegel, Miller & Jemal, 2018). Several therapeutic strategies were used for the management of PCa, including active surveillance (AS), surgery, radiotherapy (RT), hormonal therapy and chemotherapy (Litwin & Tan, 2017). Besides, 5α-reductase inhibitors (5ARIs), such as finasteride and dutasteride, play an important role in treating benign prostatic hyperplasia (BPH) by blocking 5α-reductase and inhibiting testosterone conversion to dihydrotestosterone (McConnell et al., 1992). For the prevention of PCa, the efficacy of 5ARIs has been reported in many studies (Andriole et al., 2010, 2004; Thompson et al., 2003). Results from randomized clinical trials (RCTs) showed that the incidence of PCa was lower in patients receiving 5ARIs compared with those taking placebo (Andriole et al., 2010, 2004; Thompson et al., 2003).

However, the efficacy and safety of 5ARIs in treating the PCa has not been fully researched. Previous studies reported that PCa patients treated with combined flutamide and finasteride had a significantly lower progression risk than those with flutamide alone (Banez et al., 2009). In low-risk PCa, 5ARIs led to lower rates of pathologic progression and aggressive treatments in patients receiving AS (Finelli et al., 2011; Fleshner et al., 2012). Contradictorily, recent studies demonstrated that 5ARIs did not benefit the grade reclassification and pathologic progression for PCa patients on AS (Dai et al., 2018; Ozkan et al., 2018). Adding dutasteride to bicalutamide also could not delay the disease progression in patients with non-metastatic castration-resistant prostate cancer (CRPC) (Chu et al., 2015). Therefore, the effectiveness of 5ARIs for treating PCa is still controversial. Furthermore, 5ARIs for treating PCa are not recommended in either European Association of Urology (EAU) or American Urological Association (AUA) guidelines due to inadequate high-level evidences. Although one meta-analysis was published recently exploring the association between 5ARIs therapy and PCa, it mainly focused on PCa’s incidence and only two studies were included to evaluate the effect of 5ARI therapy on PCa progression (Luo et al., 2019). We performed this meta-analysis on published literature to evaluate the effectiveness, safety, and potential advantages of 5ARIs in the treatment of PCa.

## Materials and Methods

This meta-analysis was conducted meeting the criteria of Preferred Reporting Items for Systematic Reviews and Meta-analysis (PRISMA) guidelines. Protocol of this study was registered in PROSPERO under number CRD42018109809.

### PICOS question

Patient, intervention, comparison, outcome, and study design (PICOS) guided the main question of this review: does 5ARI treatment (Intervention) improve the prognosis (PSA progression, total progression, progression-free survival time and side-effects) (Outcome) of PCa patients (Patient) compared with those who did not receive these treatments (Comparison)?

### Search strategy

Potential comparative trials studying treatment effects of 5ARIs for PCa were searched comprehensively using electronic databases, such as the Pubmed, Embase, Medline and Cochrane Library. The last search was in October, 2019. All references of included studies were also carefully checked. Language was restricted to English on eligible studies. We applied the following search terms to obtain original studies: “5alpha-reductase inhibitors” or “5-alpha reductase inhibitors” or “5ARIs” or “finasteride” or “dutasteride” in combination with “prostate cancer” or “prostate carcinoma”. Two authors (Tuo Deng and Xueming Lin) independently performed literature search and screening, quality assessment of eligible studies and data extraction. Disagreements were resolved by discussing with the third author (Guohua Zeng).

### Inclusion and exclusion criteria

Studies were included on the basis of the following criteria: (1) Studies should compare the effects of 5ARI and non-5ARI treatments for PCa patients; (2) 5ARIs should be applied after PCa diagnosis; (3) At least one prognostic outcome could be achieved, such as progression, recurrence, metastasis, progression-free survival and so on. Accordingly, studies with participants receiving 5ARIs before PCa diagnosis were excluded from this analysis. Meanwhile, abstracts, case reports, conference proceedings, non-published materials, editorials, reviews, animal experiments and repeated publications were also excluded.

### Quality assessment and data extraction

The level of evidence (LOE) of all included studies was assessed by the criteria from the Oxford Centre for Evidence-based Medicine. The quality of RCTs was evaluated using the Cochrane risk of bias tool, and the Newcastle-Ottawa Scale (NOS) was applied for the quality of non-RCTs.

Data from original studies was attentively extracted as follows: first author, year of publication, study country, population, period of recruitment, research methodology, type of PCa, tumor stage, Gleason score, previous therapy, type of 5ARIs, characteristics of participants, detailed 5ARIs treating strategy, follow-up time, and related outcomes. When incomplete data existed, we contacted authors of these studies.

### Outcome and statistical analysis

Primary outcomes of this analysis were PSA progression, total progression, progression-free survival time and side-effects. Secondary outcomes included recurrence, metastasis, biopsy reclassification during treatments and therapeutic response. PSA progression was defined as PSA value >20 ng/ml (for subjects who received no previous therapies or underwent primary RT) or >10 ng/ml (for subjects who underwent radical prostatectomy (RP) with or without salvage RT) associated with a 50% increase in PSA from baseline. Total progression referred to patients experiencing any PCa progression, including PSA progression, pathologic progression (having at least one of the predefined criteria: cancer involving ≥3 cores, Gleason pattern score ≥4, or ≥50% of any one core involved), clinical symptoms of progression, and therapeutic progression (defined as necessary post-baseline rescue therapies or medications).

Summarized odds ratios (ORs) with 95% confidence intervals (CIs) were calculated to compare dichotomous variables. Multivariable adjusted hazard ratios (HRs) of 5ARIs were also pooled for predicting progression-free survival in eligible studies. The chi-square test-based Q- and *I*^2^- statistic was used to test heterogeneity among studies. If heterogeneity was not significant with a *P* value > 0.10, the fixed-effect model was used; otherwise, the random-effect was applied. Significant results were considered with a two-sided *P* value < 0.05. For possible studies, we performed subgroup analyses according to study population, study design, tumor type, Gleason score, previous therapy, and 5ARI type. The publication bias was assessed through the inverted funnel plot visual inspection and Egger’s test. All statistical analyses were done by RevMan (version 5.3; Cochrane Collaboration, Oxford, UK) and STATA (version 13.0; StataCorp., College Station, TX, USA).

## Results

### Characteristics and quality assessment of eligible studies

**Figure 1:**
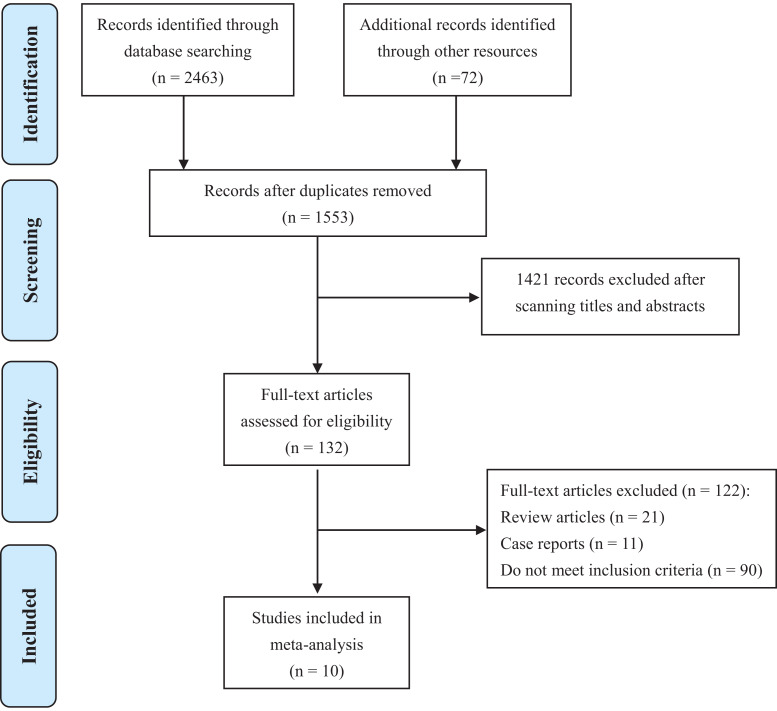
PRISMA flow diagram of study selection.

Ten studies (Andriole et al., 1995; Banez et al., 2009; Chu et al., 2015; Dai et al., 2018; Dutkiewicz, 2012; Finelli et al., 2011; Fleshner et al., 2012; Ozkan et al., 2018; Ross et al., 2012; Schroder et al., 2013) containing 2,277 PCa patients were involved in this analysis (Fig. 1). Basic characteristics were shown in Table 1. Most studies were conducted in America or Europe. All literature was published between 1995 to 2018. Among them, five were RCTs (Andriole et al., 1995; Chu et al., 2015; Dutkiewicz, 2012; Fleshner et al., 2012; Schroder et al., 2013), one was prospective cohort study (Banez et al., 2009), and other four were retrospective cohort studies (Dai et al., 2018; Finelli et al., 2011; Ozkan et al., 2018; Ross et al., 2012). Most studies focused on local PCa, while metastatic PCa (Dutkiewicz, 2012) and non-metastatic CRPC (Chu et al., 2015) were investigated in only one study respectively. Therapies that PCa patients received prior to recruitments included RP, RT, AS or no treatments. 5ARIs used in these studies were finasteride or dutasteride. In total, 694 patients were allocated to 5ARI group, and 1,583 patients belonged to the control group. Detailed treatment strategies in 5ARI and control groups for each study were also listed in Table 1.

**Table 1:** Baseline characteristics of included studies.

References	Country	Popu-lations	Recruit-ment period	Study design	LOE	Tumor type	Tumor stage	Gleason score	Previous therapy	5ARI type		No. patients	Mean age (yrs)	Mean PSA (ng/mL)	Detailed treatment strategy	Mean follow-up time (yrs)	Quality score
Andriole et al. (1995)	USA	Mixed	NA	RCT	2a	Local PCa	NA	2–10	RP or RT	Finasteride	5ARI group	54	67.8	2.2 (±2.9)	Finasteride 10 mg	2	6^#^
											no 5ARI group	66	67	2.1 (±2.6)	Placebo	2	
Banez et al. (2009)	USA	Mixed	1996–2001	PCS	2b	Local PCa	T1–T3	2–10	RP or RT	Finasteride	5ARI group	36	72.5 (65.5–76)	7.4 (±7.2)	Flutamide 125 mg twice daily and finasteride 5 mg twice daily	4.5 (0.6–7)	7*
											no 5ARI group	20	67.5 (63.5–71)	3.6 (±3.3)	Flutamide 125 mg twice daily	3.6 (2.3–7)	
Finelli et al. (2011)	Canada	Mixed	1995–2010	RCS	3	Local PCa	T1c–T2a	≤6	AS	NA	5ARI group	70	65.6 (±6.4)	5.4 (4.0–7.2)	NA	3.1 (1.6–4.9)	9*
											no 5ARI group	218	63.8 (±7.8)	4.8 (3.1–6.6)		3.8 (2.5–5.1)	
Dutkiewicz, 2012	Poland	European	NA	RCT	2a	Metastatic PCa	T3N × M1b	6–7	none	Finasteride	5ARI group	32	72 (±6.3)	17 (12–40)	Maximal Androgen Blockade administered intermittently + finasteride (Proscar) administered continuously	5	4^#^
											no 5ARI group	31	72 (±5.8)		Maximal Androgen Blockade administered intermittently		
Fleshner et al. (2012)	USA and Canada	Mixed	2006–2007	RCT	2a	Local PCa	T1c–T2a	≤6	AS	Dutasteride	5ARI group	147	65.1 (±7.1)	5.6 (±2.5)	Once-daily dutasteride 0.5 mg for 3 years	3	7^#^
											no 5ARI group	155	65.0 (±7.6)	5.8 (±2.6)	Placebo for 3 years	2.7	
Ross et al. (2012)	USA	Mixed	1994–2010	RCS	3	Local PCa	T1c	≤6	AS	Finasteride	5ARI group	47	66 (±5.6)	5.7 (±2.2)	NA	NA	8*
											no 5ARI group	540	65 (±5.8)	4.6 (±2.3)			
Schroder et al. (2013)	Mutiple countries in Europe	European	2007–2011	RCT	2a	Local PCa	T1-T3aN0M0	NA	RP or RT	Dutasteride	5ARI group	147	69.7 (±5.8)	NA	Dutasteride 0.5 mg once daily for 2 years	2	7^#^
											no 5ARI group	147	68.6 (±6.5)	NA	Placebo once daily for 2 years	1.3	
Chu et al. (2015)	USA and Canada	Mixed	2007–2013	RCT	2a	Non-metastatic CRPC	T1–T4	6–10	RP or RT or none	Dutasteride	5ARI group	62	78.9 (±5.9)	4.5 (2.0–20.0)	Bicalutamide 50 mg + Dutasteride 3.5 mg	1.5–3.5	6^#^
											no 5ARI group	65	77.6 (±7.9)	4.4 (1.7–19.0)	Bicalutamide 50 mg + Placebo	1.5–3.5	
Dai et al. (2018)	USA and Egypt	Mixed	2002–2015	RCS	3	Local PCa	T1–T2	6–7 (3 + 4)	AS	NA	5ARI group	70	66 (±7)	6.4 (±4.9)	NA	4.8 (3.8–6.3)	7*
											no 5ARI group	301	64 (±7)	5.5 (±3.4)		3.7 (2.5–6.8)	
Ozkan et al. (2018)	Turkey	European	2002–2015	RCS	3	Local PCa	≤T2c	≤6	AS	Finasteride (32%) or Dutasteride 68%)	5ARI group	29	66.5 (±6.1)	5.4 (4.3–6.5)	NA	3.3 (1.9–3.8)	8*
											no 5ARI group	40	67.7 (±8.9)	5.2 (4.0–7.1)		2 (1.4–3.1)	

**Notes:**

* Using the Newcastle-Ottawa Scale (score from 0 to 9).

# Using the Cochrane risk of bias tool (score from 0 to 7)

AS, active surveillance; CRPC, castration-resistant prostate cancer; LOE, level of evidence; PCa, prostate cancer; PCS, prospective cohort study; PSA, prostate-specific antigen; RCS, retrospective cohort study; RCT, randomized controlled trial; RP, radical prostatectomy; RT, radiotherapy; NA, not available; 5ARI, 5α-reductase inhibitor.

**Figure 2:**
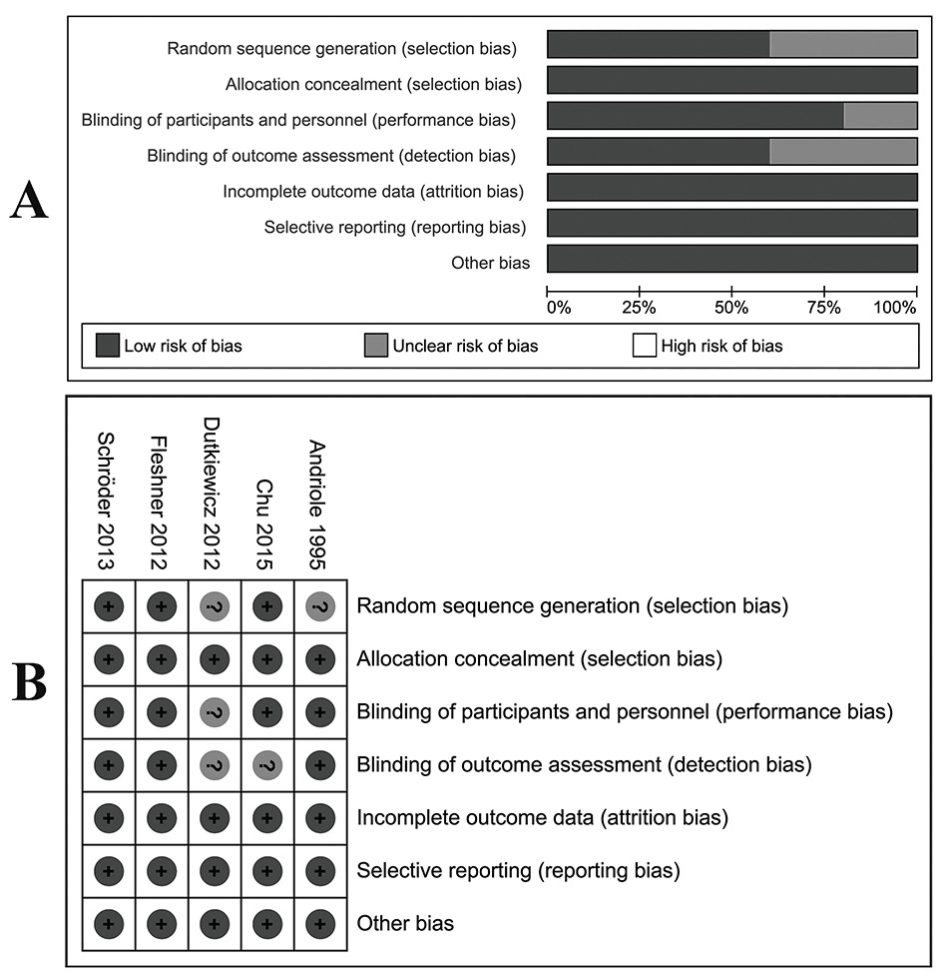
Assessment of bias risk for included RCTs. (A) Methodological quality graph: authors’ judgments about each methodological quality item presented as percentages across all included studies. (B) Methodological quality summary: authors’ judgments about each methodological quality item for each included study, “+” low risk of bias; “?” unclear risk of bias; “−” high risk of bias.

The LOE of all eligible studies was shown in Table 1. For RCTs, four (Andriole et al., 1995; Chu et al., 2015; Fleshner et al., 2012; Schroder et al., 2013) were evaluated as high-quality studies with a low risk of bias according to the Cochrane risk of bias tool. Biases of allocation concealment, incomplete outcome data and selective reporting were also at a low risk (Fig. 2). Table 2 showed the results of quality assessments of five cohort studies. All studies were relatively high in quality with seven or more stars under NOS measurements.

**Table 2:** Results of quality assessment of included cohort studies using the Newcastle-Ottawa Scale.

References	Selection				Comparability	Outcome			Scores
	Representativeness of the exposed cohort	Selection of the non-exposed cohort	Ascertainment of exposure	Demonstration that outcome of interest was not present at start of study	Comparability of cohorts on the basis of the design or analysis	Assessment of outcome	Was follow-up long enough for outcomes to occur	Adequacy of follow up of cohorts	
Banez et al. (2009)	★	★	★	★	★	★	★		7
Finelli et al. (2011)	★	★	★	★	★★	★	★	★	9
Ross et al. (2012)	★	★	★	★	★	★	★	★	8
Dai et al. (2018)	★	★	★	★	★	★	★		7
Ozkan et al. (2018)	★	★	★	★	★	★	★	★	8

**Note:**

One ★ means one point in the Newcastle-Ottawa Scale scores.

### PSA progression

**Figure 3:**
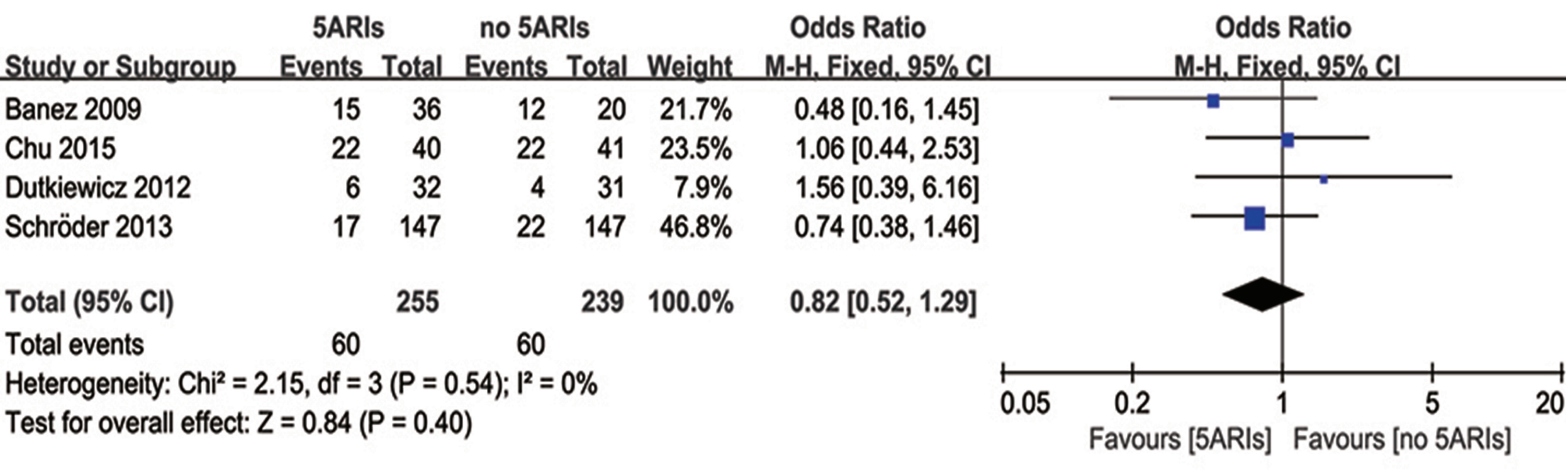
Comparison of PSA progression between prostate cancer patients with and without 5ARI treatment.

Four studies reported the PSA progression during the follow-up time. There was no heterogeneity among these four studies (*I*^*2*^ = 0%, *P* = 0.54), and no significant difference was found in PSA progression between 5ARI and control groups (OR = 0.82, 95% CI [0.52–1.29], *P* = 0.40) (Fig. 3).

In the subgroup analyses based on previous therapy, PCa patients receiving previous RP/RT (OR = 0.76, 95% CI [0.47–1.23], *P* = 0.26) or no treatments (OR = 1.56, 95% CI [0.39–6.16], *P* = 0.53) did not show significant difference in PSA progression between 5ARI and control groups; and no patient with previous AS treatments were involved in this analysis. In addition, pooled results evaluating the tumor type also indicated no significant difference in all subgroups (local PCa: OR = 0.66, 95% CI [0.37–1.17], *P* = 0.16; metastatic PCa: OR = 1.56, 95% CI [0.39–6.16], *P* = 0.53; non-metastatic CRPC: OR = 1.06, 95% CI [0.44–2.53], *P* = 0.90).

### Total progression

**Figure 4:**
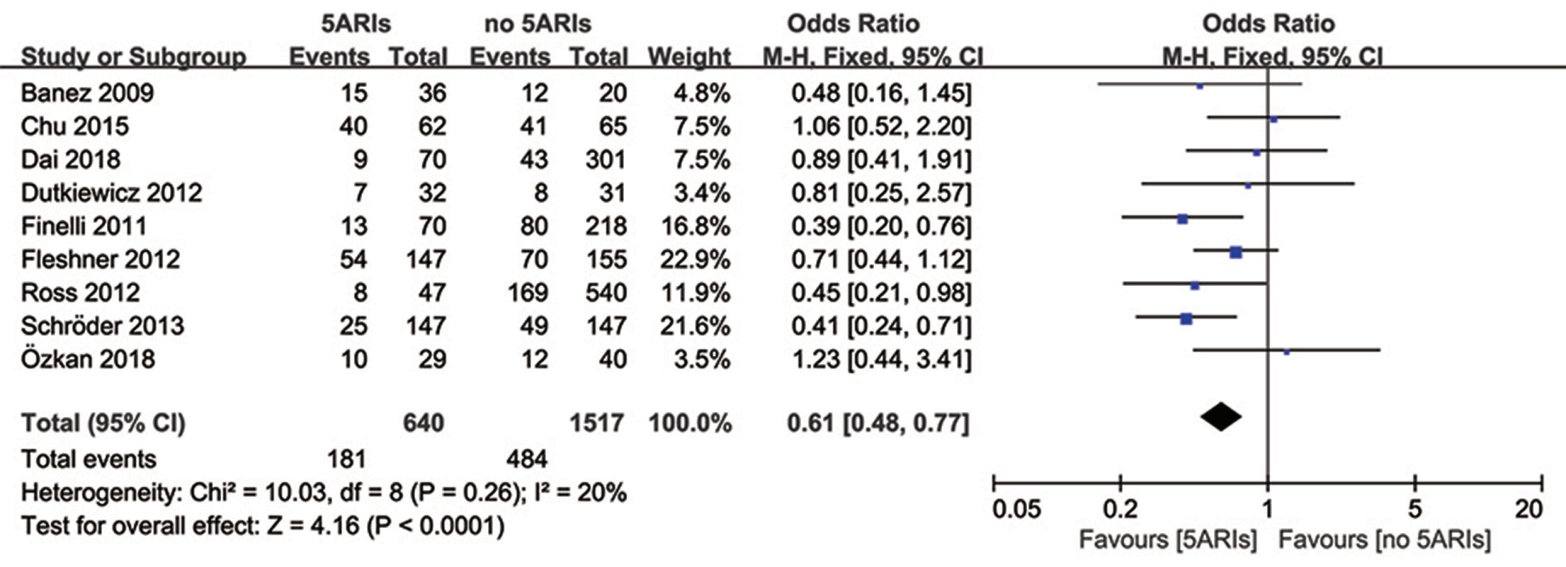
Comparison of total progression between prostate cancer patients with and without 5ARI treatment.

**Figure 5:**
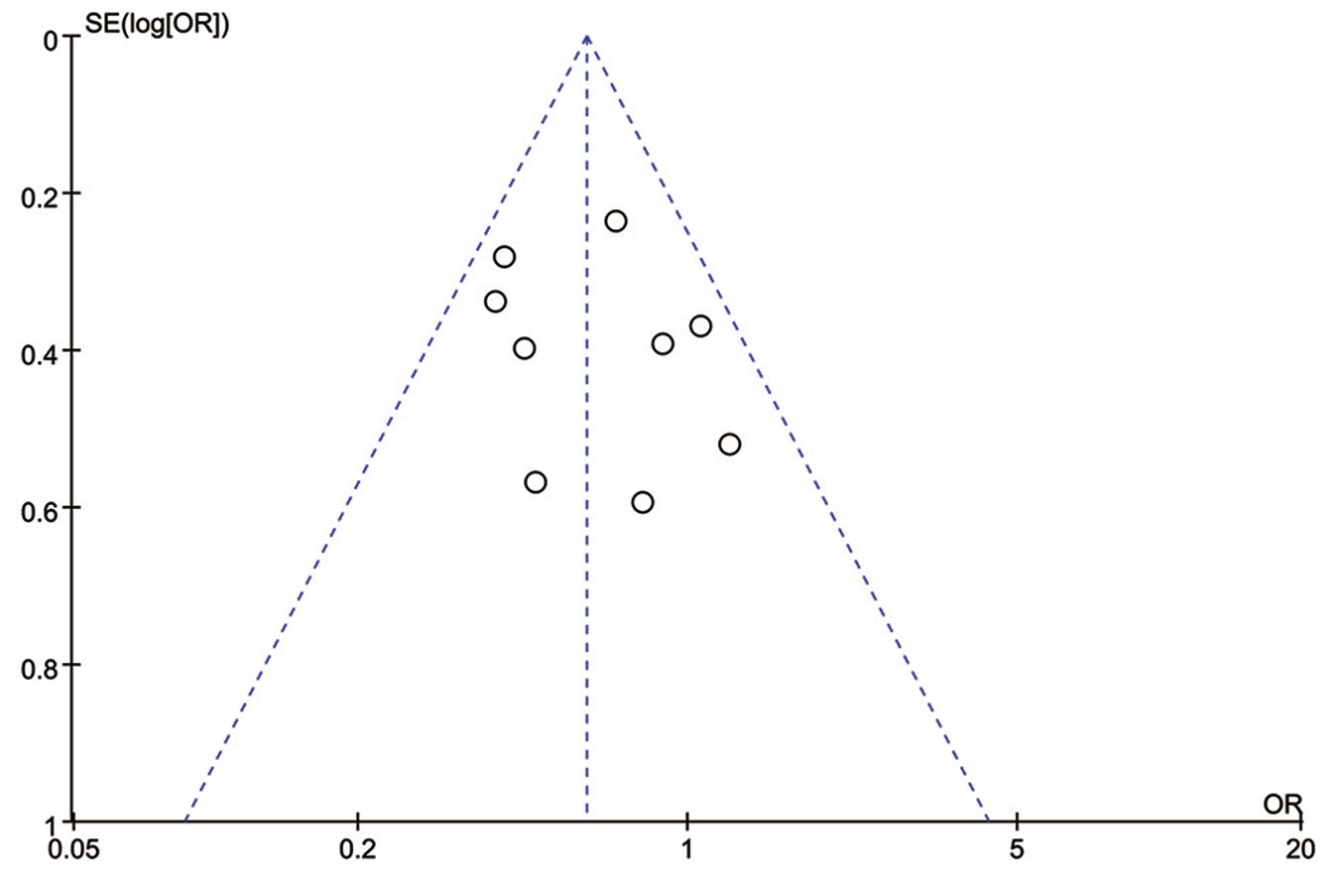
Funnel plot with pseudo 95% confidence limits of 5ARIs treatment and prostate cancer total progression.

Total progression of PCa was detected in nine included studies, and no significant heterogeneity existed (*I*^*2*^ = 20%, *P* = 0.26). Results of meta-analysis revealed that 5ARI treatment significantly reduced the total progression of PCa (OR = 0.61, 95% CI [0.48–0.77], *P* < 0.0001) (Fig. 4). No publication bias was discovered through either inverted funnel plot (Fig. 5) or Egger’s test (*t* = −0.52, *P* = 0.622).

Table 3 showed the results of subgroup analyses for total progression. It seemed that the 5ARIs were effective in decreasing PCa progression regardless of patients’ race, study design, previous therapy, and 5ARI type. However, in subgroups of tumor type and Gleason score, 5ARI treatment proved to be beneficial for patients with local PCa (OR = 0.56, 95% CI [0.44–0.73], *P* < 0.00001) and low Gleason score (≤7) (OR = 0.63, 95% CI [0.48–0.84], *P* = 0.002).

**Table 3:** Subgroup analyses of 5ARIs treatment and prostate cancer total progression.

	Subgroups	Number of included studies	No. participants	Heterogeneity		OR (95%CI)
				*I*^*2*^	*P*	
Population	European population	3	426	48%	0.14	**0.56 [0.36–0.87]**
	Mixed population	6	1,731	15%	0.32	**0.63 [0.48–0.83]**
Study design	RCT	4	786	37%	0.19	**0.64 [0.48–0.87]**
	Cohort study	5	1,371	21%	0.28	**0.57 [0.39-0.82]**
Tumor type	Local PCa	7	1,967	18%	0.30	**0.56 [0.44–0.73]**
	Metastatic PCa	1	63	NA		0.80 [0.25–2.57]
	Non-metastatic CRPC	1	127	NA		1.06 [0.52–2.20]
Gleason score	Low Gleason score (≤ 7)	6	1,680	8%	0.37	**0.63 [0.48–0.84]**
	High Gleason score (6–10)	1	127	NA		1.06 [0.52–2.20]
Previous therapy	RP or RT	2	350	0%	0.81	**0.42 [0.26–0.69]**
	AS	5	1,617	24%	0.26	**0.62 [0.46–0.84]**
5ARI type	Finasteride	3	706	0%	0.70	**0.52 [0.30–0.90]**
	Dutasteride	3	723	56%	0.10	**0.63 [0.46–0.87]**

**Notes:**

AS, active surveillance; CI, confidence interval; CRPC, castration-resistant prostate cancer; OR, odds ratio; PCa, prostate cancer; RCT, randomized controlled trial; RP, radical prostatectomy; RT, radiotherapy; NA, not applicable; 5ARI, 5α-reductase inhibitor.

Bold numbers mean the *P*-value is < 0.05.

### Progression-free survival

**Figure 6:**
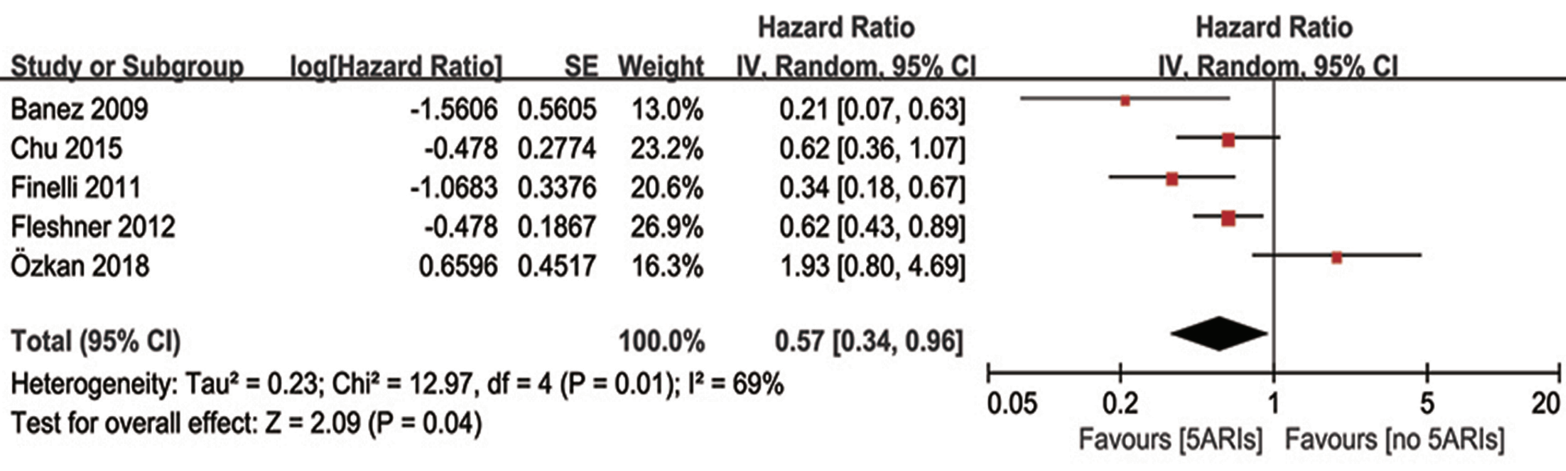
Comparison of progression-free survival time between prostate cancer patients with and without 5ARI treatment.

Multivariable adjusted HR of 5ARI treatment in predicting progression-free survival was available in five studies. Our results demonstrated 5ARIs could significantly prolonged the progression-free survival time in PCa patients (HR = 0.57, 95% CI [0.34–0.96], *P* = 0.04), with moderate heterogeneity (*I*^*2*^ = 69%, *P* = 0.01) (Fig. 6). The inverted funnel plot did not find any publication bias. In the subgroup analyses based on previous therapy, however, no significant difference was found in neither PCa patients received previous RP/RT (OR = 0.40, 95% CI [0.14–1.14], *P* = 0.09) nor AS (OR = 0.70, 95% CI [0.32–1.52], *P* = 0.36) between 5ARIs and control groups. Similarly, no significant difference was found in subgroup analyses based on tumor type (local PCa: OR = 0.56, 95% CI [0.27–1.15], *P* = 0.11; non-metastatic CRPC: OR = 0.62, 95% CI [0.36–1.07], *P* = 0.08).

### Side-effects

**Figure 7:**
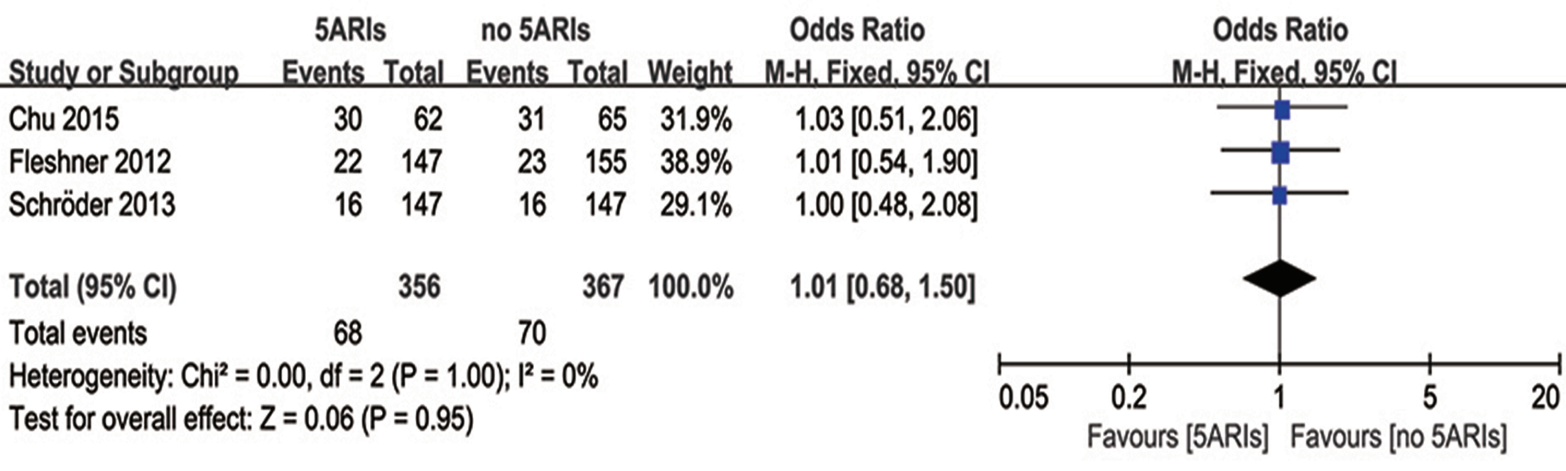
Comparison of side-effects between prostate cancer patients with and without 5ARI treatment.

Three studies compared cases with side-effects between 5ARIs and control groups. No significant difference was found in the occurrence of side-effects between two groups (OR = 1.01, 95% CI [0.68–1.50], *P* = 0.95) with no heterogeneity (*I*^*2*^ = 0%, *P* = 1.00) (Fig. 7). Publication bias was not reached from the inverted funnel plot.

### Other outcomes

Table 4 provided summarized results of outcomes explored in less than three studies. No significant differences were found in PCa recurrence, metastasis, biopsy reclassification or partial treatment response between 5ARIs and control groups. However, 5ARIs were still found to be effective in improving the complete treatment response for PCa patients (OR = 71.13, 95% CI [4.01–1262.24], *P* = 0.004).

**Table 4:** Pooled results of other outcomes.

Outcomes	Number of included studies	No. participants	Heterogeneity		OR (95%CI)
			*I*^*2*^	*P*	
Recurrence	1	120	NA		0.68 [0.27–1.70]
Metastasis	2	421	0%	0.60	0.56 [0.23–1.39]
Biopsy reclassification	2	958	0%	0.59	0.66 (0.39-1.13)
Complete response	1	63	NA		**71.13 [4.01–1,262.24]**
Partial response	1	63	NA		0.30 [0.07–1.25]

**Notes:**

CI, confidence interval; OR, odds ratio; NA: not applicable.

Bold numbers mean the *P*-value is < 0.05.

## Discussion

We concluded that 5ARIs decreased the total progression of PCa, especially for patients with local and low Gleason score (≤7) PCa. 5ARIs also prolonged the progression-free survival time and increased the rate of complete therapy response. In addition, the use of 5ARIs did not bring more side-effects to PCa patients. Above all, 5ARIs benefited more patients with local and low Gleason score (≤7) PCa, especially in delaying the disease progression. Luo et al. (2019) reported a similar meta-analysis with us recently, however, they mainly focused on the impact of 5ARIs treatment on the incidence of PCa. Although only two studies were included to evaluate the effect of 5ARIs on PCa progression, their pooled results also supported our conclusions, indicating 5ARI treatment could delay the progression of low-risk prostate cancer (Luo et al., 2019).

Androgen receptor pathway plays an important role in prostate growth and progression of prostate diseases, including BPH and PCa. Dihydrotestosterone is converted from testosterone in prostate by 5α-reductase isozymes and is more potent than testosterone. This conversion can be inhibited by 5ARIs, which decreases the level of dihydrotestosterone (McConnell et al., 1992). Thus, 5ARIs have potential effects on treating PCa. Many studies demonstrated that the use of 5ARIs reduced the risk of PCa. Preston et al. (2014) reported that 5ARIs were associated with a decrease in the incidence of overall and Gleason score ≤7 PCa based on their subgroup analysis, and did not cause the development of high-grade or lethal PCa, but the number of patients in this cohort was relatively small. Results from long-term follow-up showed that although high-grade and non-localized PCas were more common in 5ARIs group, 5ARIs had no influences on the survival outcomes (Murtola et al., 2016; Thompson et al., 2013). In general, the efficacy and safety of 5ARIs after PCa diagnosis is still unclear.

Many previous studies reported the association between 5ARIs and PCa. Dutasteride was proved to induce a dose-dependent apoptosis in androgen-sensitive human prostate cancer cells, but not in androgen-independent ones (Maria McCrohan et al., 2006). Schmidt et al. (2009) assessed the changes in gene expression in androgen-responsive xenograft tumors following dutasteride treatment. In their results, dutasteride significantly reduced the tumor size by affecting apoptosis, cytoskeletal remodeling, and cell cycle pathways. A series of studies by Wang and colleagues (Gupta et al., 2010; Masoodi et al., 2013; Pascal et al., 2015) suggested that inhibition of 5α-reductase enhanced the expression of U19/Eaf2, an androgen-regulated tumor suppressor, in LNCaP xenograft tumor during the treatment with testosterone replacement. 5ARIs can suppress the initial regrowth of regressed prostate tumors and testosterone-stimulated proliferation of LNCaP cells precultured in androgen-free media. They further used LNCaP xenograft tumor model to evaluate the effectiveness of short off-cycles during intermittent androgen deprivation therapy coupled with 5ARIs. Their results confirmed this treatment could block tumor regrowth and improve patients’ survival. Xu et al. (2006) reported that combination of dutasteride and castration had a greater tumor inhibition effect than castration monotherapy in androgen-responsive LNCaP cancers, but not in androgen-independent PC-3 human prostatic cancer xenografts. Another study pointed out dutasteride combined with anti-androgen drugs suppressed ERG fusion-positive prostate cancer cell growth and reduced the tumor burden (Ateeq et al., 2012). All the above studies demonstrated possible mechanisms and potential effects of PCa treated by 5ARIs.

The low-risk localized PCa is very common and it is tested more frequently under PSA screening (Cooperberg et al., 2004). Kearns et al. (2019) reported that continuous use of 5ARIs on patients who were enrolled in AS programs for PCa reduced the rate of RP or any other curative treatments, but did not influence the reclassification. Five included studies in our meta-analysis were performed among patients on AS. Our result indicated that 5ARIs reduced the progression and prolonged the progression-free survival time, especially in patients with local PCa or Gleason score ≤7. It was suggested that these patients on AS benefited more from 5ARIs treatment.

Finasteride is a selective type 2 5α-reductase inhibitor, while dutasteride is a dual 5α-reductase inhibitor. Based on previous studies, the level of 5α-reductase type 2 was lower and type 1 was higher in PCa tissues compared with those in normal prostates (Thomas et al., 2008; Xu et al., 2006). Both types were elevated in high-grade PCa tissues compared with low-grade ones (Thomas et al., 2008). Ateeq et al. (2012) reported that type 1 and type 3 5α-reductase were expressed more in metastatic tumor tissues than in benign and local ones. Consequently, the reactivities to 5ARIs were different due to expression levels of 5α-reductase. It might be one of the reasons for various results in these studies. Further studies are still needed to determine how to identify PCa patients who will benefit from 5ARIs.

Some limitations also should be considered in this meta-analysis. First, due to very limited numbers of studies that focused on single topics such as single study design type, tumor type, 5ARI type and previous therapy, we had to combine these different types of studies together, which may bring heterogeneity and potential bias to our results. Thus, our results should be treated cautiously. Second, there was not enough data to conduct subgroup analyses on doses and treatment duration; what’s more, the numbers of included studies in other subgroup analyses were also small. Third, the sample size of some studies was small, which may impact the quality of data analyses. Additionally, studies in languages other than English, with incomplete data, or not published, were excluded in our analysis, which may introduce the enrollment bias.

## Conclusions

Our study demonstrates that 5ARI treatment can benefit patients with local and low Gleason score (≤7) PCa, especially in delaying the disease progression. More studies with larger sample size and comprehensive study design are still needed to further verify our outcomes.

## Supplemental Information

10.7717/peerj.9282/supp-1 Supplemental Information 1 PRISMA checklist.

10.7717/peerj.9282/supp-2 Supplemental Information 2 Detailed search strategy in Pubmed.

10.7717/peerj.9282/supp-3 Supplemental Information 3 Meta-Analysis Rationale.

## Abbreviations

PCa: prostate cancer
AS: active surveillance
RT: radiotherapy
5ARI: 5α-reductase inhibitor
BPH: benign prostatic hyperplasia
RCT: randomized clinical trial
CRPC: castration-resistant prostate cancer
EAU: European Association of Urology
AUA: American Urological Association
LOE: level of evidence
NOS: Newcastle-Ottawa Scale
RP: radical prostatectomy
OR: odds ratio
CI: confidence interval
HR: hazard ratio

## Additional Information and Declarations

### Data Availability

The following information was supplied regarding data availability: All data generated or analyzed in this study are available in the “Results”, Tables 1–4 and Figs. 1–7.
