# Supplementary material for: Prostate cancer patients can benefit from 5-alpha-reductase inhibitor treatment: a meta-analysis
Source: PeerJ. 2020 Jun 1;8:e9282. doi: 10.7717/peerj.9282 (PMC7271889; doi:10.7717/peerj.9282)
Supplement: Supplemental Information 1 [file peerj-08-9282-s001.doc]

| **Section/topic** | **#** | **Checklist item** | **Reported on page #** |
| --- | --- | --- | --- |
| TITLE | | | 1 |
| Title | 1 | Identify the report as a systematic review, meta-analysis, or both. | 1 |
| ABSTRACT | | | 2 |
| Structured summary | 2 | Provide a structured summary including, as applicable: background; objectives; data sources; study eligibility criteria, participants, and interventions; study appraisal and synthesis methods; results; limitations; conclusions and implications of key findings; systematic review registration number. | 2 |
| INTRODUCTION | | | 3-4 |
| Rationale | 3 | Describe the rationale for the review in the context of what is already known. | 3: The efficacy and safety of 5ARIs in treating the PCa has not been fully researched. |
| Objectives | 4 | Provide an explicit statement of questions being addressed with reference to participants, interventions, comparisons, outcomes, and study design (PICOS). | 4: We performed this meta-analysis on published literature to evaluate the effectiveness, safety, and potential advantages of 5ARIs in the treatment of PCa. |
| METHODS | | | 4-6 |
| Protocol and registration | 5 | Indicate if a review protocol exists, if and where it can be accessed (e.g., Web address), and, if available, provide registration information including registration number. | 4: Protocol of this study was registered in PROSPERO under number CRD42018109809. |
| Eligibility criteria | 6 | Specify study characteristics (e.g., PICOS, length of follow-up) and report characteristics (e.g., years considered, language, publication status) used as criteria for eligibility, giving rationale. | 4: The sub-section “Inclusion and exclusion criteria” described this item. |
| Information sources | 7 | Describe all information sources (e.g., databases with dates of coverage, contact with study authors to identify additional studies) in the search and date last searched. | 4: A comprehensive electronic literature search using the Pubmed, Embase, Medline and Cochrane Library was conducted up to October, 2019. |
| Search | 8 | Present full electronic search strategy for at least one database, including any limits used, such that it could be repeated. | 4: Search terms were “‘5alpha-reductase inhibitors’ or ‘5-alpha reductase inhibitors’ or ‘5ARIs’ or ‘finasteride’ or ‘dutasteride’” in combination with “‘prostate cancer’ or ‘prostate carcinoma’”. Full electronic search strategy in Pubmed was shown in supplymentary materia 2. |
| Study selection | 9 | State the process for selecting studies (i.e., screening, eligibility, included in systematic review, and, if applicable, included in the meta-analysis). | 4: Studies were selected according to the inclusion and exclusion criteria. Details can be found in the sub-section “Inclusion and exclusion criteria”. |
| Data collection process | 10 | Describe method of data extraction from reports (e.g., piloted forms, independently, in duplicate) and any processes for obtaining and confirming data from investigators. | 5: Data from all eligible studies was attentively extracted. Authors of some studies were contacted to obtain incomplete data. |
| Data items | 11 | List and define all variables for which data were sought (e.g., PICOS, funding sources) and any assumptions and simplifications made. | 5: Primary outcomes of this analysis were PSA progression, total progression, progression-free survival time and side-effects. Secondary outcomes included recurrence, metastasis, biopsy reclassification during treatments and therapeutic response. Detailed definitions can be found in the first paragraph of the sub-section “Outcome and statistical analysis”. |
| Risk of bias in individual studies | 12 | Describe methods used for assessing risk of bias of individual studies (including specification of whether this was done at the study or outcome level), and how this information is to be used in any data synthesis. | 5: The quality of RCTs was evaluated using the Cochrane risk of bias tool, and the Newcastle-Ottawa Scale (NOS) was applied for the quality of non-RCTs. |
| Summary measures | 13 | State the principal summary measures (e.g., risk ratio, difference in means). | 5: Summarized odds ratios (ORs) with 95% confidence intervals (CIs) were calculated to compare dichotomous variables. |
| Synthesis of results | 14 | Describe the methods of handling data and combining results of studies, if done, including measures of consistency (e.g., I2) for each meta-analysis. | 5-6: Details can be found in the second paragraph of the sub-section “Outcome and statistical analysis”. |

Page 1 of 2

| Section/topic | # | Checklist item | Reported on page # |
| --- | --- | --- | --- |
| Risk of bias across studies | 15 | Specify any assessment of risk of bias that may affect the cumulative evidence (e.g., publication bias, selective reporting within studies). | 5: The publication bias among eligible studies was assessed through the inverted funnel plot visual inspection and Egger’s test. |
| Additional analyses | 16 | Describe methods of additional analyses (e.g., sensitivity or subgroup analyses, meta-regression), if done, indicating which were pre-specified. | 5: Subgroup analyses were performed according to study population, study design, tumor type, Gleason score, previous therapy, and 5ARIs type. |
| RESULTS | | | 6-8 |
| Study selection | 17 | Give numbers of studies screened, assessed for eligibility, and included in the review, with reasons for exclusions at each stage, ideally with a flow diagram. | 6: 10 studies containing 2277 PCa patients were included in this analysis (Figure 1). |
| Study characteristics | 18 | For each study, present characteristics for which data were extracted (e.g., study size, PICOS, follow-up period) and provide the citations. | 6: Basic characteristics of all eligible studies were shown in Table 1. |
| Risk of bias within studies | 19 | Present data on risk of bias of each study and, if available, any outcome level assessment (see item 12). | 6: Figure 2 showed the quality of all included RCTs; and Table 2 showed the quality of all included non-RCTs. |
| Results of individual studies | 20 | For all outcomes considered (benefits or harms), present, for each study: (a) simple summary data for each intervention group (b) effect estimates and confidence intervals, ideally with a forest plot. | 6-8: Details can be found in the “Results” section. |
| Synthesis of results | 21 | Present results of each meta-analysis done, including confidence intervals and measures of consistency. | 6-8: Details can be found in the “Results” section. |
| Risk of bias across studies | 22 | Present results of any assessment of risk of bias across studies (see Item 15). | 7-8: No obvious publication bias was detected. |
| Additional analysis | 23 | Give results of additional analyses, if done (e.g., sensitivity or subgroup analyses, meta-regression [see Item 16]). | 7-8: Subgroup analyses were performed according to study population, study design, tumor type, Gleason score, previous therapy, and 5ARIs type. |
| DISCUSSION | | | 8-10 |
| Summary of evidence | 24 | Summarize the main findings including the strength of evidence for each main outcome; consider their relevance to key groups (e.g., healthcare providers, users, and policy makers). | 8: The first paragraph of “Discussion” section summarized the main findings of this study. |
| Limitations | 25 | Discuss limitations at study and outcome level (e.g., risk of bias), and at review-level (e.g., incomplete retrieval of identified research, reporting bias). | 10: The last paragraph of “Discussion” section stated the limitations of this study. |
| Conclusions | 26 | Provide a general interpretation of the results in the context of other evidence, and implications for future research. | 10: Can be found in the “Conclusion” section. |
| FUNDING | | | 11 |
| Funding | 27 | Describe sources of funding for the systematic review and other support (e.g., supply of data); role of funders for the systematic review. | 11 |

*From:*  Moher D, Liberati A, Tetzlaff J, Altman DG, The PRISMA Group (2009). Preferred Reporting Items for Systematic Reviews and Meta-Analyses: The PRISMA Statement. PLoS Med 6(7): e1000097. doi:10.1371/journal.pmed1000097

For more information, visit: **www.prisma-statement.org**.

Page 2 of 2
