# Supplementary material for: Prostate cancer patients can benefit from 5-alpha-reductase inhibitor treatment: a meta-analysis
Source: PeerJ. 2020 Jun 1;8:e9282. doi: 10.7717/peerj.9282 (PMC7271889; doi:10.7717/peerj.9282)
Supplement: Supplemental Information 2 [file peerj-08-9282-s002.docx]

**Detailed search strategy in Pubmed:**

((prostate carcinoma[All Fields] OR prostate cancer[All Fields]) AND (((((5-alpha reductase inhibitors[All Fields] OR 5ARI[All Fields]) OR 5alpha-reductase inhibitors[All Fields]) OR 5ARIs[All Fields]) OR finasteride[All Fields]) OR dutasteride[All Fields]))) NOT review[Filter] AND ("humans"[MeSH Terms] AND "male"[MeSH Terms])
