# Supplementary material for: Prostate cancer patients can benefit from 5-alpha-reductase inhibitor treatment: a meta-analysis
Source: PeerJ. 2020 Jun 1;8:e9282. doi: 10.7717/peerj.9282 (PMC7271889; doi:10.7717/peerj.9282)
Supplement: Supplemental Information 3 [file peerj-08-9282-s003.docx]

**Meta-Analysis Rationale**

**1. The rationale for conducting this meta-analysis:**

Prostate cancer (PCa) is the second most common cancer and the fifth leading cause of cancer mortality in men all over the world. 5α-reductase inhibitors (5ARIs), such as finasteride and dutasteride, can block 5α-reductase, inhibit testosterone converting to dihydrotestosterone, and then play an important role in treating benign prostatic hyperplasia (BPH). For the prevention of PCa, the efficacy of 5ARIs has been reported in many studies. Results from randomized clinical trials (RCTs) showed that the incidence of PCa was lower in patients receiving 5ARIs compared with those taking placebo.

However, the efficacy and safety of 5ARIs in treating the PCa has not been fully researched. Previous studies reported that PCa patients treated with combined flutamide and finasteride had a significantly lower progression risk than those with flutamide alone. In low-risk PCa, the use of 5ARIs was associated with lower rates of pathologic progression and aggressive treatments in patients undergoing AS. Contradictorily, some recent studies showed that 5ARIs had no obvious impacts on the grade reclassification and pathologic progression in PCa patients on AS. Adding dutasteride to bicalutamide did not delay the disease progression in patients with non-metastatic castration-resistant prostate cancer (CRPC) either. Therefore, the effectiveness of 5ARIs for treating PCa is still controversial.

Although one meta-analysis published recently explored the association between 5ARIs therapy and incidence and progression of PCa, they mainly focused on the incidence of PCa, only 2 studies were included to evaluate the effect of 5ARIs therapy on PCa progression. Consequently, we performed this meta-analysis on published literature to evaluate the effectiveness, safety, and potential advantages of 5ARIs in the treatment of PCa.

**2. The contribution that the meta-analysis makes to knowledge in light of previously published related reports, including other meta-analyses and systematic reviews:**

Previously, only one meta-analysis published explored the association between 5ARIs therapy and incidence and progression of PCa. However, they mainly focused on the incidence of PCa, only 2 studies up to July 2018 were included to evaluate the effect of 5ARIs therapy on PCa progression. In our meta-analysis, a total of 10 studies up to October 2019 were included to evaluate the effectiveness and safety of 5ARIs in the treatment of PCa. Previous meta-analysis conducted by Luo et al only focused on cancer-specific mortality and progression of PCa based on few eligible studies. Our study focused on PSA progression, total progression, progression-free survival time, side-effects, recurrence, metastasis, biopsy reclassification during treatments and therapeutic response of PCa. Thus, our meta-analysis is more comprehensive and updated, and can provide more information.
